# The effectiveness and cost-effectiveness of lay counsellor-delivered psychological treatments for harmful and dependent drinking and moderate to severe depression in primary care in India: PREMIUM study protocol for randomized controlled trials

**DOI:** 10.1186/1745-6215-15-101

**Published:** 2014-04-02

**Authors:** Vikram Patel, Benedict Weobong, Abhijit Nadkarni, Helen A Weiss, Arpita Anand, Smita Naik, Bhargav Bhat, Jesina Pereira, Ricardo Araya, Sona Dimidjian, Steven D Hollon, Michael King, Jim McCambridge, David McDaid, Pratima Murthy, Richard Velleman, Christopher G Fairburn, Betty Kirkwood

**Affiliations:** 1 Sangath Centre, Alto-Porvorim, 403521 Goa, India; 2 Faculty of Epidemiology and Population Health, London School of Hygiene and Tropical Medicine, London, UK; 3 Kintampo Health Research Centre, Ghana Health Service, Kintampo, Ghana; 4 Department of Psychology and Neuroscience, University of Colorado, Boulder, CO, USA; 5 Department of Psychology and Human Development, Vanderbilt University, Nashville TN, USA; 6 Department of Mental Health Sciences, University College, London, UK; 7 Personal Social Services Research Unit, London School of Economics and Political Science, London, UK; 8 National Institute of Mental Health and Neurosciences, Bangalore, India; 9 Department of Psychology, University of Bath, Bath, UK; 10 Department of Psychiatry, University of Oxford, Oxford, UK

**Keywords:** Alcohol use disorders, Depression, Low- and middle-income countries, Non-specialist health workers, Psychological treatments

## Abstract

**Background:**

The leading mental health causes of the global burden of disease are depression in women and alcohol use disorders in men. A major hurdle to the implementation of evidence-based psychological treatments in primary care in developing countries is the non-availability of skilled human resources. The aim of these trials is to evaluate the effectiveness and cost-effectiveness of two psychological treatments developed for the treatment of depression and alcohol use disorders in primary care in India.

**Methods/design:**

This study protocol is for parallel group, randomized controlled trials (Healthy Activity Program for moderate to severe depression, Counselling for Alcohol Problems for harmful and dependent drinking) in eight primary health centres in Goa, India. Adult primary care attendees will be screened with the Patient Health Questionnaire for depression and, in men only, the Alcohol Use Disorders Identification Test for drinking problems. Screen-positive attendees will be invited to participate; men who screen positive for both disorders will be invited to participate in the Counselling for Alcohol Problems trial. Those who consent will be allocated in a 1:1 ratio to receive either the respective psychological treatment plus enhanced usual care or enhanced usual care only using a computer generated allocation sequence, stratified by primary health centre and, for depression, by sex. The enhanced usual care comprises providing primary health centre doctors with contextualized World Health Organization guidelines and screening results. Psychological treatments will be delivered by lay counsellors, over a maximum period of three months. Primary outcomes are severity of disorder and remission rates at three months post-enrolment and, for the Counselling for Alcohol Problems trial, drinking and the impact of drinking on daily lives. Secondary outcomes include severity of disorder and remission rates at 12 months, disability scores, suicidal behaviour and economic impact, and cost-effectiveness at three and 12 months. 500 participants with depression and 400 participants with harmful drinking will be recruited. Primary analyses will be intention-to-treat.

**Discussion:**

These trials may offer a new approach for the treatment of moderate-severe depression and drinking problems in primary care that is potentially scalable as it relies on delivery by a single pool of lay counsellors.

**Trial registration:**

Both trials are registered with the International Society for the Registration of Clinical Trials (Healthy Activity Programme registration number ISRCTN95149997; Counselling for Alcohol Problems registration number ISRCTN76465238).

## Background

Psychological treatments (PTs) are ‘talking’ treatments in which a therapist aims to modify ways of thinking, feeling, behaving and relating with others with the goal of facilitating recovery from distressing mental phenomena. The vast majority of people with mental disorders who live in low- and middle-income countries (LMICs) do not have access to any form of treatment, particularly PTs. Two major barriers to making treatments accessible are the lack of skilled human resources for delivering treatments and concerns regarding the context and generalizability of treatments developed in ‘western’ cultural settings. The goal of PREMIUM, a **Pr**ogram for **E**ffective **M**ental Health **I**nterventions in **U**nder-resourced Health Syste**m**s, is to implement a PT development and evaluation methodology that will lead to effective PTs for mental disorders that are culturally appropriate, feasible, acceptable and affordable in under-resourced settings. A core element of this methodology is a long-term vision of ultimate scalability of treatments by emphasizing their delivery by the same pool of lay counsellors working in routine primary health care settings.

This methodology is being applied for the treatment of two mental disorders affecting adults: depression and alcohol use disorder (AUD). These two disorders have been chosen for the following reasons. First, they are the two leading causes of the global burden of mental disorders; depression is the leading mental disorder affecting women, AUD is the leading mental disorder affecting men [1]. Both disorders are associated with profound adverse social impacts on the persons affected and their families. Second, although effective pharmacological treatments exist for the severe forms of both disorders, PTs are recommended as first-line treatments in LMICs [2,3]. The primary target groups will be moderate to severe depression and harmful drinking (HD). This is because the World Health Organization (WHO) treatment guidelines for primary care advocate the use of brief structured PTs for moderate to severe depression and HD [4]. Antidepressants are also advocated for moderate to severe depression but these benefit only a third to half of patients with depression [5] and a recent trial of collaborative care for common mental disorders in primary care in India observed low adherence [6]. Furthermore, PTs are associated with lower relapse rates when compared with antidepressant medication [7] and a recent UK trial demonstrated that adding PT to routine primary care for treatment-resistant depression greatly enhanced recovery rates [5]. ‘Brief interventions’ are the cornerstone for the management of HD [8]; they are as effective as more extended interventions, can be effectively delivered in primary care and are twice as likely to reduce drinking compared with no intervention [9-12]. Limited evidence exists in support of brief interventions for reducing drinking in patients with more severe forms of AUD, namely alcohol dependence (AD). However, there is some evidence that shows that brief interventions lead to reduced drinking in patients with AD [13,14] and it is well established that rates of engagement with addictions treatment services can be enhanced [9,13,15-17].

The vast majority of trials of PT for depression and AUD are from specialist settings in high-income countries. The generalizability of these findings to LMICs is compromised by several major contextual factors, such as variations in the explanatory models of these disorders, ways of coping and dealing with them, marked variability in access to specialist services, and distinct socio-economic determinants (such as literacy) [18,19]. There is a growing evidence base testifying to the effectiveness of approaches that address these challenges, notably the systematic adaptation of PTs to suit the local context and task-sharing their delivery to appropriately trained and supervised lay and community health workers in primary care and community settings [20]. There is emerging evidence testifying to the cost-effectiveness of task-sharing of PTs to non-specialist health workers (NSHW) compared with enhanced usual care [21]. However, a recent systematic review observed that task-sharing is associated with a number of barriers, primarily a lack of resources [22], and generating further evidence on its cost-effectiveness is a key strategy to address this barrier. Identifying effective PTs delivered by NSHWs was ranked amongst the leading research priorities for global mental health in a recent systematic priority-setting exercise [23].

PREMIUM is a Wellcome Trust-funded programme that seeks to develop and evaluate PTs for priority mental disorders for delivery by NSHWs in routine health care settings in India. The programme has systematically developed PTs for moderate to severe depression and HD and AD (Chowdhary *et al*., in preparation; Nadkarni *et al*., in preparation). PREMIUM will now implement two concurrent randomized controlled trials to evaluate each of these treatments, delivered by the same NSHWs (referred to as lay counsellors).

### Objectives and hypotheses

The objectives of the two trials are to evaluate the effectiveness and cost-effectiveness of the Healthy Activity Program (HAP), for adults with moderate to severe depression, and the Counselling for Alcohol Problems (CAP), for adults with HD or AD, delivered by the same pool of lay counsellors in primary care in Goa, India. The primary analysis group for the CAP trial are participants with HD. The primary hypotheses are that the PT intervention in addition to enhanced usual care (EUC) will be superior to EUC alone in reducing the severity of symptoms and in increasing remission rates in participants with depression and HD at three months post-enrolment. In addition, we hypothesize that the CAP will reduce the physical, social, intrapersonal, impulsive and interpersonal consequences of alcohol use at three months in participants with HD. Secondary hypotheses are that the PT intervention in addition to EUC will be superior to EUC alone in reducing disability, suicidal behaviour and intimate partner violence, and that it will be cost-effective from a health systems perspective. That is, it would have a gain in quality-adjusted life years of no more than the annual per capita gross domestic product in India. Further to this, from a societal perspective, the intervention will be dominant over EUC, with both a reduction in costs and superior outcomes.

We further hypothesize that the PT intervention will increase activation (HAP trial) and uptake of detoxification services (CAP trial). Table 1 provides a summary of the trials outcomes.

**Table 1:** Primary and secondary outcomes of the PREMIUM trials

**Outcome**	**Source of data (see Table **5**for details on each measure)**		**End-point**	**Analysis group**
	**Healthy Activity Program**	**Counselling for Alcohol Problems**		
Severity of symptoms	Beck depression inventory-II	Time line follow back	3^a^ and 12 months	Depression^a^, HD^a^, AD
Remission	Patient health questionnaire	Alcohol use disorders identification test	3^a^ and 12 months	Depression^a^, HD^a^, AD
Consequences of alcohol use	NA	Short inventory of problems	3^a^ and 12 months	HD^a^, AD
Disability levels	WHO disability assessment schedule	WHO disability assessment schedule	3 and 12 months	Depression, HD, AD
Costs of illness	Client service receipt inventory	Client service receipt inventory	Over 12 months	Depression, HD, AD
Suicidal behaviour	Patient Health Questionnaire-9 and additional questions on suicide attempts	Patient Health Questionnaire-9 and additional questions on suicide attempts	3 and 12 months	Depression, HD, AD
Experience of intimate partner violence	Questionnaire on intimate partner violence	Questionnaire on intimate partner violence	3 and 12 months	Depression
Level of behavioural activation	Adapted version of the behavioural activation for depression scale - short form	-	3 and 12 months	Depression
Uptake of detoxification services	NA	Client service receipt inventory	3 and 12 months	AD

^a^Primary hypotheses. AD, alcohol dependence; HD, harmful drinking; NA, not applicable.

## Methods/design

### Setting

The trials will be conducted in eight primary health centres (PHC) in the district of North Goa, a state on the west coast of India. The publicly funded PHC is the first port of call in India for people who wish to seek health care in the public system.

### Design

We will carry out a parallel arm randomised controlled trial with equal allocation of participants between arms.

### Participants and procedures

**Figure 1:**
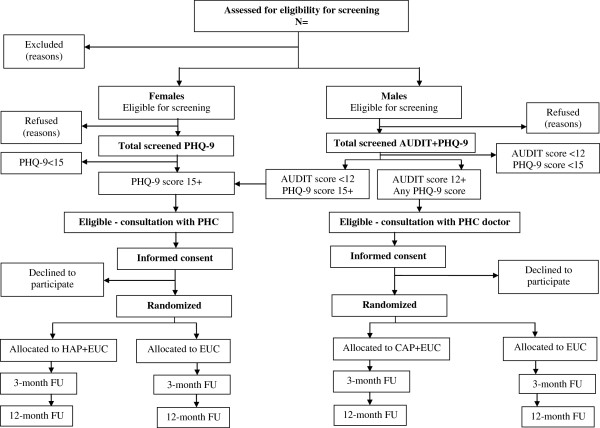
**PREMIUM trials flow chart.** AUDIT, Alcohol Use Disorders Identification Test; Counselling for Alcohol Problems; HAP, Healthy Activity Program; PHQ-9, Patient Health Questionnaire-9; PHC, Primary Health Clinic; FU, Follow Up.

The flow chart (Figure 1) shows the process of recruitment and follow-up of participants in the trials. The HAP trial will include participants of both genders, and the CAP trial will only include male participants because HD and AD are rare in women in India [24]. Eligible patients will be invited to participate in either the HAP or CAP trial as appropriate by the health assistant after the patient’s consultation session with the PHC doctor. After provision of written and verbal information, consent will be sought. A record of the age, educational attainment, marital status, gender, screening questionnaire score and reason for refusal will be maintained for those who do not consent. Eligible patients who are willing to participate in the trial but are unable to complete the informed consent procedure due to time constraints will be invited to return on another occasion, or followed-up by the health assistant at their homes or a convenient location to complete the procedure. Such patients will be allowed a period of two weeks to confirm their participation in the trials. Recruitment will be censored to a maximum of the first two to three eligible participants per day (irrespective of the type of disorder) to ensure uniform staggering across all PHCs (which vary in size of patient attendance) and to avoid over-loading of counsellors.

#### Inclusion criteria

Eligible participants will be PHC attendees aged 18 to 65 years who are intending to reside at the same address in the catchment area of the PHC for at least 12 months. This age range was chosen because persons younger than 18 years require parental consent for participation in research and our pilot study showed that those over the age of 65 had particular difficulties in engaging with the PT. Participants will be included if they screen positive on the Patient Health Questionnaire-9 (PHQ-9) and/or the Alcohol Use Disorders Identification Test (AUDIT), and if they give informed consent.

#### Exclusion criteria

Pregnant women, patients who need urgent medical attention (defined as needing emergency treatment or in-patient admission), patients who are unable to communicate clearly (for example, due to a speech or hearing disability or inability to comprehend one of the programme’s four languages: Konkani, Hindi, Marathi or English), and patients who are intoxicated at the point of assessing inclusion and exclusion criteria will not be eligible. These criteria will be determined by trained health assistants at the PHC.

#### Identification of the target mental health conditions

Trained health assistants will interview consecutive PHC attendees at the out-patient department who meet the inclusion criteria. To detect depression they will use the PHQ-9, a nine-item questionnaire that has previously been validated for use in the study setting [25]; the cut-point of 14 selected is the most accurate for detection of moderate to severe depression [26] and has a high positive predictive value for depressive disorder defined by the International Classification of Diseases revision 10 [27]. To detect HD or AD**,** they will use the AUDIT, a 10-item screening questionnaire for AUD, developed by the WHO [28]. The AUDIT has been validated and used in cross-national studies, including in India [29], and has been field-tested in the study setting [30]. The AUDIT allows for varying the cut-off score depending on the country’s drinking patterns, the alcohol content of standard drinks, and the nature of the screening program. For the purpose of this study the threshold score will be 12. The AUDIT has been adapted to cover a three-month period to be consistent with the three-month outcome assessment, as in other studies [31].

The order of assessments for male patients will be the AUDIT first, followed by the PHQ-9; those who screen positive for both disorders will be invited to participate in the CAP trial, following the guidelines for the treatment of co-morbid depression and HD and AD [32]. Patients who screen negative and re-enter the PHC will not be offered the opportunity for re-screening until three months after their initial screening.

### Baseline assessments

After consent has been obtained, the health assistant will collect the following data on potential moderators of treatment effect: age group, gender, marital status, employment status [33], duration of the illness [33] (for HAP), readiness to change drinking behaviour [34] (for CAP), severity of depression or drinking [35], and the patient’s expectations in the effectiveness of PTs for their health problem [36]. All assessments will be audio-taped (with permission), and the tapes randomly selected for review by the supervisor for quality assurance.

### Randomization

Randomization will be carried out after completion of the baseline assessments. The randomization list will be generated by a statistician independent of the trial. The list will be stratified by PHC and sex (for the HAP trial) using randomly sized blocks of four to six (two to four for men in the HAP trial). The randomization code will be concealed using sequentially numbered opaque sealed envelopes to maximise allocation concealment [37]. The envelopes will be prepared at the London School of Hygiene and Tropical Medicine (LSHTM) by personnel independent of the trial and shipped to the data manager at the trial site. We will ensure fidelity of treatment allocation is not compromised by using differently coloured envelopes for the two trials, each of which will have a sticker with clinic number, gender (for HAP) and trial ID number. The envelopes will be taped down for extra security. Inside will be a plain piece of card folded in two, and inside the card will be a sticker with the ID number and allocation. A daily check by the data manager will be performed to evaluate if allocations done are consistent with the allocation code.

### Sample size estimation

The sample size estimations are made on the following assumptions: patients are randomized within each of the eight clinics; one counsellor per PHC; an intra-cluster correlation of 0.04 (this is based on the MANAS trial [6] and may be conservative, as the CoBalT trial found within-therapist clustering of only 0.0027 [5]); a loss to follow-up of 15% over three months (conservative, based on the MANAS trial in Goa, which was 13% over six months [6]); and equally sized groups (1:1 allocation ratio).

Based on these assumptions, we aim to recruit 500 participants with depression for the HAP trial and 600 participants with HD or AD for the CAP trial, to yield the required sample size of 400 patients with HD (AUDIT scores of 12 to 19; those who score > 19 are categorized as AD). The power we will have to detect effect sizes for the outcomes for analyses of primary and secondary analysis groups are specified in Tables 2, 3 and 4.

**Table 2:** Power calculations for the Healthy Activity Program trial

**Outcome**	**Total sample size**	**Number per arm**	**Number of clinics**	**Treatment arm**	**Enhanced usual care arm**	**SD**	**Effect size**	**Power**
BDI-II mean score (primary)	500	250	8	16.8	17.9^a^	2.8^a^	0.4^c^	86%
	500	250	8	16.65	17.9^a^	2.8^a^	0.45^c^	93%
	500	250	8	16.5	17.9^a^	2.8^a^	0.5^c^	97%
	500	250	8	18.9	24.5^b^	10.7^b^	0.5	98%
Remission (PHQ-9 < 10) (primary)	500	250	8	62%	44%^d^	-	-	81%
	500	250	8	65%	44%^d^	-	-	92%
WHO DAS mean score (secondary)	500	250	8	15.0	17.0^e^	5.0^e^	0.4	87%
	500	250	8	14.5	17.0^e^	5.0^e^	0.5	97%

^a^Based on pilot PREMIUM data, where BDI score was 17.9 with SD 2.8. ^b^Based on the COBALT trial [5]. The mean BDI-II score at six months was 24.5 (SD 13.1) in the usual care arm and 18.9 (SD 14.2) in the intervention arm (among participants with a BDI > 14 at baseline). The study reported an effect size of 0.53, based on the pooled SD of baseline BDI-II score (SD = 10.7). ^c^Effect size of 0.4 to 0.5 is based on the recent meta-analyses of behavioural therapy for depression, which found a summary effect size of 0.42 for depressive symptoms based on low intensity interventions (internet-based or guided interventions with limited support by a health professional) [38] and of 0.7 (95% CI: 0.39, 1.00) for all behavioural interventions [39]. Two further trials found effect sizes of 0.53 [5] for a cognitive behavioural therapy-intervention on BDI score and 0.76 [40] for behavioural activation on BDI score. ^d^Proportion recovered from any common mental disorder among baseline depression cases in MANAS in the enhanced usual care arm at three months. ^e^Based on 12-month WHO-DAS scores among those who had moderate or severe common mental disorders at baseline in the MANAS trial. BDI, Beck’s Depression Inventory; PHQ-9, Patient Health Questionnaire-9; WHO DAS, World Health Organization Disability Assessment Schedule.

**Table 3:** Power calculations for the Counselling for Alcohol Problems trial

**Outcome**	**Total sample size**	**Number per arm**	**Number of clinics**	**Treatment arm**	**Enhanced usual care arm**	**Effect size**	**Power**
Mean standard ethanol content consumed in past two weeks	400	200	8			0.4^a^	82%
	400	200	8			0.45	93%
	400	200	8			0.5	97%
AUDIT score < 8 at 12 months	400	200	8	60%	40%^b^	-	84%
	400	200	8	68%	40%^b^	-	99%

^a^Based on the effect size in the meta-analysis by Vasilaki *et al*. which found an effect size of 0.4 (95% CI: 0.36, 0.44) for Motivational Interviewing versus no treatment in reducing alcohol consumption among non-dependent drinkers [41]. ^b^Based on the randomized controlled trial by Bager *et al*., which found abstinence rates of 68% versus 40% two months after randomisation [42].

**Table 4:** Power calculations for the Counselling for Alcohol Problems trial on available sample sizes for sub-group analyses

**Sub-group**	**Outcome**	**Sample size**	**Power**	**Effect size**
Harmful drinking and alcohol dependence	Mean standard ethanol content consumed in past two weeks	638	0.50	99%
			0.40	92%
			0.33	80%
			0.40	63%
			0.33	48%
Alcohol dependence	Mean standard ethanol content consumed in past two weeks	238	0.50	86%
			0.40	69%

### The PREMIUM interventions

#### The psychological treatments

The PTs were developed in a systematic process that built upon the experiences of the investigators in adapting mental health interventions for use in under-resourced and socio-culturally diverse contexts [43-50]. The key principles of this approach were to dismantle evidence-based PTs and combine them with strategies identified as being useful in the local context. Evidence-based PTs were identified based on the WHO Mental Health Gap Action Programme (mhGAP) guidelines for use in primary care [4]. Contextually appropriate strategies were identified through a review of explanatory models, PT studies and qualitative studies with persons with the target disorders, their care-givers and health care providers (including alternative and religious healers) about key outcomes and coping strategies. The strategies were collated and the list reduced by merging those that were similar. Strategies that were rated by local mental health providers and NSHWs as being acceptable, safe and feasible for delivery by NSHWs in primary care were taken to the next stage of synthesis into a formal PT in intervention development workshops. This process led to the identification of behavioural activation and motivational enhancement therapy as the theoretical framework for the emerging PTs for depression and HD or AD respectively. The next phase of treatment development involved the development and adaptation of PT manuals based on the source behavioural activation manual [51] and motivational enhancement therapy manual [52], and by testing the delivery of the PTs to patients with the target disorders both by specialists and lay counsellors. The PTs were finally subjected to a pilot study in which lay counsellors delivered them to patients in PHCs; modifications were made at this stage to enhance acceptability, feasibility and scalability, notably through the inclusion of home-based delivery, use of pictorial patient resource materials, strategies to encourage the involvement of a significant other in the treatment, and task-sharing the supervision of lay counsellors to peer-groups. In addition, a generic counselling skills manual was used to train counsellors in core skills required for the delivery of both PTs. These manuals of the treatments are available on a database on the Sangath website [53].

The resulting PT for depression is the HAP. HAP comprises up to eight sessions delivered in three flexible phases over two to three months with each session lasting between 30 and 45 minutes. The optimal number of sessions for HAP are five or six, at which point the pilot study showed the largest reduction in depression scores; however, a few patients needed up to eight sessions to achieve this outcome. Patients are encouraged to complete the first session on the day of recruitment; those unable to complete a full session are offered an abbreviated session. The core strategies of HAP are behavioural assessment and self-monitoring, psychoeducation about the relationship between activity and mood, activity structuring and scheduling, and problem-solving. The CAP uses content derived from motivational interviewing along with other behavioural and cognitive components. CAP has three phases, and is delivered flexibly over one to four sessions (30 to 45 minutes each) over six to eight weeks. The optimal number of sessions is two, with a maximum of four sessions for the small proportion of patients who are either at the more severe end of the spectrum or do not achieve treatment goals. The core strategies of CAP are detailed assessment, personalised feedback, evoking commitment to change and development of a change plan, drink refusal skills, managing drinking urges, management of emotions, problem-solving skills, and relapse prevention. In addition, patients with AD will receive a structured referral to detoxification services.

Both PTs will be delivered on an individual basis either at the PHC, the patient’s home or a convenient place suggested by the patient, or on the telephone. Any participant receiving the maximum number of stipulated sessions of the PT or completing treatment goals in fewer sessions will be discharged. Participants receiving HAP who do not respond to the PT at the end of eight sessions will receive a referral to psychiatric services. Any participants missing three consecutive scheduled sessions will be considered as a treatment drop-out. However, participants who re-engage at any point during the trial will be offered the opportunity to continue from the last session.

##### Counsellors

The PTs will be delivered by lay counsellors who are members of the local community, are above 18 years of age, have completed at least high school education, do not have professional mental health training, and have an expressed desire to help people with mental health problems. Trainee counsellors were recruited by placing advertisements in newspapers and through word of mouth, and selected based on their performance in a structured interview and role play. Post selection, the trainees underwent a three-week participatory workshop covering both PTs. Trainees who met competency standards (based on role play and multiple choice questions) progressed to the pilot study. Training was conducted by five local specialists who were previously trained by international experts and continued to receive once monthly supervision via Skype. In the pilot study the trainee counsellors delivered the PT to eligible patients in PHCs. Supervision was carried out both in individual and weekly group format by the local specialists. Therapy quality was assessed mainly through rating of audio-taped sessions using a specially developed scale, the Quality of HAP and Quality of CAP adapted from the Counselling Skills Scale [54], the Quality of Behavioural Activation Scale (Dimidjian S, Hubley A, Martell CR, Herman-Dunn R, Dobson KS: **Quality of behavioral activation scale (Q-BAS): **Unpublished) and the Motivational Interviewing Target Scheme [55]. As the trainee counsellors gained experience in delivering the intervention, the supervision format evolved from expert led (that is, local mental health professionals skilled in the delivery of the PT) to peer-led group supervision. Only trainees who achieved competence, as assessed by standardised role plays and therapy quality assessments, were selected to deliver the PTs in the trials. Each PHC will have one counsellor, with a pool of back-up counsellors in the event of counsellors leaving or if weekly load of patients exceeds counsellor limit.

##### Supervision

Supervision during the trial will be primarily in a peer-led group format with supervision groups held once a week in the field office or a PHC. Supervision will comprise two types of activities: first, assessment of the quality of sessions (using the quality rating scales) based on selected audio-recordings of sessions and, provision of peer feedback; and second, discussion of any difficult cases. If treatment-related issues cannot be resolved in the peer group they will be discussed with experts. In addition, individual supervision will take place twice a month on-site (that is, at the respective PHC) by an expert who will review individual patient progress and quality of documentation, assess any patient safety issues, and address any site- or counsellor-specific practical difficulties or concerns.

### Enhanced usual care

Usual care in primary care for depression and HD or AD in India is, in effect, no care at all. This has been confirmed in the study setting during the pilot study. This is primarily because most cases are not diagnosed and, amongst those who are, most do not receive either antidepressants or PT. In the PREMIUM trials, usual care will be enhanced by providing the screening results to the primary care physician; providing a contextualized version of the mhGAP guidelines [4] for the target disorders to the primary care physician, including guidelines on when and where to refer patients for psychiatric care.

This EUC is the most intensive model of primary care for both target disorders that may be envisaged in the foreseeable future in India or other LMICs, and is the one that is currently recommended by the WHO’s mhGAP [56].

### Minimization of contamination

Contamination of the EUC arm will be minimized by masking the PHC doctor to the allocation status of the patient and not offering formal training in the PTs to any of the PHC staff. If there are any clinical issues arising from the counselling that need PHC doctor involvement, the counsellor will advise the patient to speak to the doctor about this, or will consult the expert supervisor for guidance.

### Outcome evaluation

Outcome data will be collected at three months and 12 months post-enrolment. The three-month outcome is the primary end-point for both trials as the PTs delivery will be completed and we would expect the optimal effect of the treatment. The 12-month end-point is included to evaluate the sustainability of the effect of the intervention. The outcome assessment measures with the specific outcomes for each trial are summarized in Table 5. The primary outcome measures for the HAP trial are the Beck’s Depression Inventory and the PHQ-9, and for the CAP trial, the Time Line Follow Back, the Short Inventory of Problems and the AUDIT. The additional costs of delivering PTs and EUC, impacts on the use of health care services, lost time from everyday usual activities including paid work, as well as time and monetary costs to families will be assessed using data collected with a tailored version of the Client Service Receipt Inventory.

**Table 5:** PREMIUM outcome assessments

**Instrument**	**Trial**	**Description**	**Outcome**	**Contextual validity**
Beck Depression Inventory-II	HAP	21-item questionnaire assessment of depressive symptoms assessed on a scale of 0 to 3.	Mean total score	Widely used measure for evaluating depression in trials, including in India [57,58].
Time Line Follow Back	CAP	Calendar tool supplemented by memory aids to obtain retrospective estimates of daily drinking over a specified time period.	Alcohol consumed in past two weeks (g)	Validated instrument [59] used in India [60].
AUDIT	CAP	10-item questionnaire with three questions on the amount and frequency of drinking, three questions on alcohol dependence and four on problems caused by alcohol.	Mean AUDIT score	Validated with primary health care patients in six countries [28] and used in study setting [61].
			Remission (AUDIT score < 8)	
Short Inventory of Problems	CAP	15-item questionnaire which assesses physical, social, intrapersonal, impulsive and interpersonal consequences of alcohol consumption.	Adverse consequences of alcohol consumption	Validated instrument [62] and used in India [63]. Translated into Konkani using standardized procedure followed by piloting.
PHQ-9	HAP CAP	Nine-item questionnaire assessment of depressive symptoms assessed on a scale of 0 to 3.	Prevalence of moderate-severe depression; mean total score.	Validated in primary care and Konkani version validated in Goa [25].
WHO Disability Assessment Schedule	HAP CAP	12-item questionnaire for measuring functional impairment over the previous 30 days. In addition, two items assess number of days the person was unable to work in the previous 30 days.	Total disability score; quality adjusted life years; number of days out of work.	Validated for international use [64] and used in previous trials in Goa [6,65].
Client Service Receipt Inventory	HAP CAP	Questionnaire to collect data on the utilization and costs of health care and lost productivity (including that of care-givers).	Costs of illness (direct and indirect)	Previously used in trials in the study setting [66,67] and elsewhere in India [65,21].
Violence and suicidal behaviour	HAP CAP	Item 9 of the PHQ-9 with additional questions on suicide attempts and IPV.	Suicide plans and attempts	Based on interviews used in earlier studies in Goa [68].
			Experience of IPV (for HAP trial only)	
			Perpetration of IPV (for CAP trial only)	
Abbreviated Behavioural Activation for Depression Scale - short form	HAP	Adapted version of the Behavioural Activation for Depression Scale - short form [69].	Indicators of behavioural activation	Translated into Konkani using standardized procedure followed by piloting.

AUDIT, Alcohol Use Disorders Identification Test; CAP, Counselling for Alcohol Problems; HAP, Healthy Activity Program; IPV, intimate partner violence, PHQ-9, Patient Health Questionnaire-9.

### Masking of outcome assessments

Baseline assessments will be carried out by the health assistants in the PHC before randomization. The three- and 12-month outcome assessments will be carried out by an independent team of field workers who have no contact with the PHCs and who will be entirely community based (that is, assessments will be done at home, to minimize the risk of unmasking). The intervention and outcome evaluation teams will not have any interactions during the trial and will maintain separate physical location and administrative management. The evaluators and participants will be told that we are evaluating two different interventions and that there is clinical equipoise about whether one is better than the other. Primary outcome measures will be completed at the first contact, reducing the likelihood of it being affected by unmasking during the assessment.

### Fidelity assessments

Two types of indicators will be collated to evaluate the fidelity of the delivery of the PTs, namely, their quantity and quality. Quantity indicators collated through counsellor case records will measure the number, mode of delivery (that is, home, PHC, telephone), and duration of PT sessions. Quality indicators will be assessed through ratings of 10% of audio-recording transcripts of all sessions by independent experts blind to outcome data using the respective PT quality assessment scales.

### Nested qualitative study

The aim of the qualitative study is to explore patient perceptions of the quality of the care received and any beneficial or adverse effects, satisfaction with care, and the impact of their health problems on their daily lives, economic productivity and lives of family members. Participants will be purposively recruited after completing the 12-month outcome assessments by the independent statistician (to maintain blinding) to ensure balance of arms, recovery status and PHCs. We expect to carry out in-depth interviews with up to 40 participants from each trial, within one to two weeks of completion of the secondary end-point quantitative assessments. In-depth interviews will be conducted until data saturation is reached. All interviews will be audio-taped. Subsequently, the memos (reflective notes about the interviews) and field notes written by the interviewers will be attached to the main text of the interviews. The qualitative researchers will be separate from the quantitative outcome assessment team.

### Data management

Three types of quantitative data will be collected: baseline, intervention process and outcome assessment. All baseline and outcome data (with the exception of a part of the Client Service Receipt Inventory) will be captured electronically using tablet computers, as will process data from lay counsellors. The data will be remotely uploaded as comma separated values (CSV) files on the main data server running online using the customized STAR software program [70], which is compliant with Good Clinical Practice (including a date- and time-stamp for original data entry, and an audit trail documenting any subsequent changes). PT process data and therapy quality data will be collected in paper form; these will be manually entered and stored as CSV files using the same data collection platform used for the electronic data. Range and consistency checks will be performed at weekly intervals separately for each data source. Queries identified will be resolved promptly by the trial management team, and the database updated, maintaining the audit trail. All data will be kept in separate databases and only merged into a master database after data collection is completed and each individual database is locked. All data is backed-up on external hard disks on a daily basis. Access to pre-locked data will be password-protected at multiple levels and no member of the trial team apart from the data manager and independent statistician will have access to these passwords. After the dataset is locked, it will remain password-protected and trial investigators will have access to the datasets. Qualitative data will be collected using digital recorders together with written field notes and memos. The former will be transcribed in the language of the interview, anonymized but linked with the trial ID and then translated for analysis. A similar procedure will be followed for the written data. Digital recordings will be stored in a secure, password-protected folder. For all data, a separate file linking names and trial IDs will be kept and password-protected.

### Analysis

Quantitative analyses will be carried out using Stata (version 13). Below is a summary of our approach to the analysis. A detailed analysis plan will be agreed with the Data Safety and Monitoring Committee towards the end of the trial and before any analysis is undertaken.

#### Descriptive analyses

Initial analyses will compare baseline characteristics of individuals who consented and did not consent, and participants who did and did not complete outcome assessments respectively. Baseline characteristics of enrolled participants will be compared between treatment arms. Findings will be reported as per the CONSORT guidelines [71], including a trial flow chart. This will include total adult PHC attendees within period of screening, total assessed for inclusion and exclusion criteria, number of patients meeting inclusion or exclusion criteria, number screened for eligibility, number consenting to enter the trials, and number refusing or excluded (with reasons). The number continuing through the trials, actively withdrawing, and passively lost to follow-up will be shown by arm. The outcome measures will be summarised at recruitment, at three-month and 12-month follow-up by intervention arm, and overall. These will be summarised by means (standard deviation), medians (interquartile range), or numbers and proportions as appropriate by key relevant subgroups (such as age, gender and baseline outcome score). For continuous outcomes, histograms will also be plotted within each arm to assess normality and whether any transformation is required.

#### Outcome analyses

The primary analyses will be intention-to-treat at the three-month end-points adjusted for baseline values (where assessed), regardless of adherence to the PT. The size of the trial and the randomization design should ensure balance between arms. However, this will be checked and adjustments included for any *a priori* defined potential confounding variables for which randomization did not achieve reasonable balance between the two arms at baseline. PHC will be adjusted for as a fixed effect in the analysis to allow for within-PHC clustering. There will usually be only one counsellor per PHC so we will not additionally adjust for counsellor variation. Analyses of outcome will be conducted using logistic regression for binary outcomes (for example, percentage with depression, percentage with HD), and linear regression for continuous outcomes (for example, depression scores). Effect sizes will be reported as crude and adjusted relative risks estimated using the marginal standardization technique with 95% confidence intervals for the ratios estimated via the delta method [72] for binary outcomes; and mean differences and standardised mean differences with 95% confidence intervals for continuous outcomes. Missing outcome data will be imputed using multiple imputation, implemented in Stata [73]. Given that we have only two follow-up time points (three and 12 months), the analyses will be conducted and interpreted separately for each of these time points. No interim analyses of outcomes are planned.

#### Moderator analyses

A moderator analysis will be conducted to help clarify for whom and under what circumstances (moderators) each of the PT treatments work. We will assess modification of treatment effect by *a priori* defined modifiers (age group, gender, marital status, employment status, chronicity of illness (for depression), readiness to change drinking (for HD or AD) severity of depression or drinking, and patient expectations), by fitting relevant interaction terms and testing for heterogeneity of treatment effects in regression models.

#### Compliance analysis

As we expect a proportion of our participants to have poor compliance to the PTs, we will undertake Complier’s Average Causal Effect analyses, which estimates the effect of the PT on the participants who received it in full as intended by the original randomization [74].

#### Economic evaluation

Cost-effectiveness analysis and cost utility analysis will be conducted assessing the incremental cost-effectiveness of PT versus EUC in both trials. The cost-effectiveness analysis will make use of the primary clinical outcome data collected in the trials, while quality of life impacts estimated by transforming data collected using the WHO Disability Assessment Schedule will be used for cost utility analysis. The economic analysis will be conducted from both a health system perspective and a wider perspective incorporating societal impacts that include costs to families and impacts on economic productivity. The costs of the PTs will be estimated by deriving a monetary value for each component of the treatment based on actual time and resources required for delivery and costs incurred, and applying these to each individual based on the process indicators that reflect the actual uptake of the treatment. Other health care, patient- or family-borne resource use and costs, including any informal caregiver time costs, and productivity losses will be computed and compared at three and 12 months, and subsequently related to changes in health outcome. In the event that dominance is not shown, that is, the PT is more effective but the costs are also more than the EUC group, incremental cost-effectiveness ratios will be computed, together with their confidence intervals (using bootstrapping techniques to overcome expected skewness of cost data). Cost-effectiveness acceptability curves will also be derived in order to show the probability of any cost-effective advantages for the PTs at a range of 'willingness to pay' threshold levels, for both the health system and the broader societal perspectives.

#### Qualitative study analysis

Thematic analysis will be carried out using appropriate software. Two researchers will first read and familiarize themselves with the data. Then they will select 10 interviews and inductively generate initial codes through reading the interview transcript. Based on the coded data and the original research questions, the codes will be defined and collated into potential themes, and the code book refined. Inter-rater reliability will be tested by double-coding 10 randomly selected interview transcripts and the code book finalized. The researchers will then code the entire dataset. Vivid and compelling examples of narrative extracts will be selected, analysed and related to the research questions. Deviant case analysis will be done to examine narratives that are not consistent with the themes.

### Ethical considerations

The trial protocol has been granted ethical approval from the Sangath and LSHTM Institutional Review Boards. The LSHTM is the trial sponsor. Written (or witnessed, if the participant is illiterate) informed consent will be mandatory for enrolment. All consent procedures will be audio-taped, with the patient’s approval, for quality assurance. Separate consent will be taken for participation in the qualitative study. All participants will be able to access EUC, which represents a higher quality of care than what is currently available and is consistent with the type of care currently envisaged in India's District Mental Health Program. We will protect the confidentiality of personal data principally through procedures to separate study data and participant identifiable data. Quantitative data gathered in the tablets for each participant at baseline will retain personal identification items to minimize errors in transcribing identities, but these will be removed before transferring the data to Stata for analysis. We will monitor the occurrence of three specific serious adverse events (SAEs) - death, suicide attempt, and unplanned hospitalization from any cause, will be recorded at three- and 12-month outcome assessments. Their reporting and appropriate responses will be governed by the standard operating procedure approved by the Data Safety and Monitoring Committee, which sets out the *a priori* criteria for unblinding of adverse events (that is, a statistically significant difference in prevalence between the two arms at *P* <0.01). SAEs will be compiled by the data manager at the end of the week during outcome follow-ups, and the report shared with the principal investigator and a psychiatrist independent of the trials. The independent psychiatrist will make contact with the respondent within 24 hours of receiving the SAE report, to arrange a convenient time and place to complete a detailed interview either on phone or face to face and offer any necessary intervention. A blinded summary of SAEs will be sent to the Data Safety Monitoring Committee and Institutional Review Boards on a quarterly frequency. Patients in the HAP trial who remain symptomatic at the end of the trial will be offered free specialist care services from the independent psychiatrist.

### Trial management

Three committees will monitor the progress of the trial (Table 6). Trial monitoring will comprise the collation and reporting of routine trial process indicators and adverse events*.* Summary statistics and graphs showing trends over time will be compiled for the process indicators, and reported on a weekly basis to the Trial Management Committee and monthly to all members of the Trial Steering Committee. The Data Safety and Monitoring Committee will receive three-monthly reports of SAEs, the trial flow charts, and tables showing baseline comparability of patient characteristics between arms.

**Table 6:** Trial management committees

**Committee**	**Role**	**Members**	**Frequency of meeting**
Trial Management Committee (TMC)	To monitor all aspects of the conduct and progress of the trial, ensure that the protocol is adhered to and take appropriate action to safeguard participants and the quality of the trial itself.	• Principal investigator	Weekly
		• Trial manager	
		• Intervention team leaders	
		• Outcome evaluation coordinator	
		• Project coordinator	
		• Data manager	
Trial Steering Committee (TSC)	To provide overall supervision of the trial and ensure that it is being conducted in accordance with the protocol and the relevant regulations. The TSC should approve the trial protocol and any protocol amendments and provide advice to the TMC on all aspects of the trial. Decisions about continuation or termination of the trial or substantial amendments to the protocol are finally the responsibility of the TSC.	• Independent chairperson (Lakshmi Vijayakumar, a psychiatrist and trialist from Chennai)	Six-monthly
		• Co-investigators (Betty Kirkwood, Christopher G Fairburn, Helen Weiss and Michael King)	
		• Members of the TMC	
Data Safety Monitoring Committee (DSMC)	The DSMC will review the accruing trial serious adverse event reports to assess whether there are any safety issues that should be brought to participants’ attention or any reasons for the trial not to continue. It is the only body that makes recommendations to unblind data and makes further recommendations to the TSC.	• Sunita Bandewar (anthropologist with expertise in research ethics),	Six-monthly
		• Soumitra Pathare (psychiatrist with expertise in mental health law and human rights, member of national Mental Health Policy Group)	
		• Paulomi Sudhir (clinical psychologist)	
		• Nikhil Gupte (biostatistician, runs a clinical trials unit)	

## Discussion

The PREMIUM trials will extend evidence on the effectiveness of PTs when delivered by NSHWs in LMIC contexts [20]. The trials, which address two leading causes of the mental health-related global burden of disease, are designed to be used in routine primary health care settings, employing minimum exclusion criteria for participation, using the same pool of affordable and available human resources, and include a comprehensive economic assessment. These factors all point to the potential scalability of the findings. The main limitation of our trial design is that we are not using diagnostic interviews to characterize our participants or the outcomes; however, these are not practical in the context of the trial and not generalizable in the real-world of primary health care. The delivery agent of the PTs is not a routine health care worker but a counsellor specifically employed by the programme, and thus the trial findings will need to be interpreted cautiously in terms of their scalability. Finally, while we have argued for the rationale of using the same pool of counsellors to deliver both PTs, there is an obvious risk that this design may reduce their effectiveness in delivering each PT with optimal quality and fidelity. Ultimately, we anticipate that the long-term impact of the evidence generated by PREMIUM will be to contribute to closing the treatment gap for mental disorders in low-resourced settings through scaling-up effective PTs for delivery by NSHWs by offering a range of practical tools (such as treatment manuals, patient resource materials and quality assessment tools) to complement the WHO’s mhGAP guidelines. In doing so, new treatments will be developed with potential applicability not only in LMICs but in well-resourced contexts as well, as the human resource costs of mental health care spiral in all countries.

## Trials status

Enrolment for the trials begun on 28 October 2013. Based on our experiences with the pilot study, we expect approximately 4% prevalence of depression and HD (based on screening over 14,000 adult PHC attendees) and a 50% participation rate. Thus, with eight PHCs, each enrolling three participants per week for either trial, we expect to complete recruitment of our target samples in 12 months (that is, by the end of October 2014) and complete the primary end-point three-month outcome evaluation by the end of January 2015. The final 12-month outcome evaluation will be completed by the end of October 2015. In the event that one trial attains the expected sample size ahead of the other, recruitment will still continue till the other trial achieves its target. This is to ensure consistency in trial design and that counsellors are able to see similar groups of patients so that they do not behave differently.

## Abbreviations

AD: alcohol dependence; AUD: alcohol use disorders; AUDIT: Alcohol Use Disorders Identification Test; CAP: Counselling for Alcohol Problems; EUC: enhanced usual care; HAP: Healthy Activity Programme; HD: harmful drinking; LSHTM: London School of Hygiene and Tropical Medicine; LMICs: low- and middle-income countries; mhGAP: Mental Health Gap Action Programme; NSHWs: non-specialist health workers; PHC: primary health centre; PHQ-9: patient health questionnaire; PREMIUM: **Pr**ogram for **E**ffective **M**ental Health **I**nterventions in **U**nder-resourced Health Syste**m**s; PTs: psychological treatments; SAE: serious adverse events; WHO: World Health Organization; WHO DAS: WHO Disability Assessment Schedule.

## Competing interests

The authors declare that they have no competing interests.

## Authors’ contributions

VP: conception, design, analysis of pilot data and manuscript writing. BW and AN: pilot data collection, analysis of pilot data and critical revision of the manuscript. HAW: design, analysis of pilot data and critical revision of the manuscript. AA, SN, BB, JP, SD, SH: pilot data collection and critical revision of the manuscript. RA, RV: design and critical revision of the manuscript. MK, JM: design and critical revision of the manuscript. DM, PM: pilot data analysis and critical revision of the manuscript. CF, BRK: conception, design and critical revision of the manuscript. All authors read and approved the final manuscript.

## References

[B1] Lopez AMC Ezzati M Jamison D Murray C Global Burden of Disease and Risk Factors 2006 Washington: Oxford University Press and the World Bank

[B2] Benegal V Chand PK Obot IS Packages of care for alcohol use disorders in low- and middle-income countries PLoS Med 2009 6 e1000170 10.1371/journal.pmed.1000170 19859536 PMC2761617

[B3] Patel V Simon G Chowdhary N Kaaya S Araya R Packages of care for depression in low- and middle-income countries PLoS Med 2009 6 e1000159 10.1371/journal.pmed.1000159 19806179 PMC2747016

[B4] WHO mhGAP Intervention Guide for Mental, Neurological and Substance Use Disorders in non-Specialized Health Settings 2010 Geneva, Switzerland: WHO 23741783

[B5] Wiles N Thomas L Abel A Ridgway N Turner N Campbell J Garland A Hollinghurst S Jerrom B Kessler D Kuyken W Morrison J Turner K Williams C Peters T Lewis G Cognitive behavioural therapy as an adjunct to pharmacotherapy for primary care based patients with treatment resistant depression: results of the CoBalT randomised controlled trial Lancet 2013 381 375 384 10.1016/S0140-6736(12)61552-9 23219570

[B6] Patel V Weiss HA Chowdhary N Naik S Pednekar S Chatterjee S De Silva MJ Bhat B Araya R King M Simon G Verdeli H Kirkwood BR Effectiveness of an intervention led by lay health counsellors for depressive and anxiety disorders in primary care in Goa, India (MANAS): a cluster randomised controlled trial Lancet 2086–2095 2010 376 10.1016/S0140-6736(10)61508-5 PMC4964905 21159375

[B7] Vittengl JR Clark LA Dunn TW Jarrett RB Reducing relapse and recurrence in unipolar depression: a comparative meta-analysis of cognitive-behavioral therapy's effects J Consult Clin Psychol 2007 75 475 488 17563164 10.1037/0022-006X.75.3.475 PMC2630051

[B8] Heather N Breaking new ground in the study and practice of alcohol brief interventions Drug Alcohol Rev 2010 29 584 588 10.1111/j.1465-3362.2010.00204.x 20973840

[B9] Bien TH Miller WR Tonigan JS Brief interventions for alcohol problems: a review Addiction 1993 88 315 335 10.1111/j.1360-0443.1993.tb00820.x 8461850

[B10] Kahan M Wilson L Becker L Effectiveness of physician-based interventions with problem drinkers: a review Can Med Assoc J 1995 152 851 859 7697578 PMC1337758

[B11] Wilk A Jensen N Havighurst T Meta-analysis of randomized control trials addressing brief interventions in heavy alcohol drinkers J Gen Intern Med 1997 12 274 283 10.1007/s11606-006-5063-z 9159696 PMC1497107

[B12] Kaner E Beyer F Dickinson H Pienaar E Campbell F Schlesinger C Heather N Saunders J Burnan B Effectiveness of brief alcohol interventions in primary care populations Cochrane Database Syst Rev 2007 2 CD004148 10.1002/14651858.CD004148.pub3 17443541

[B13] Liu SI Wu SI Chen SC Huang HC Sun FJ Fang CK Hsu CC Huang CR Yeh HM Shih SC Randomized controlled trial of a brief intervention for unhealthy alcohol use in hospitalized Taiwanese men Addiction 2011 106 928 940 10.1111/j.1360-0443.2010.03330.x 21205050

[B14] Field CA Caetano R The effectiveness of brief intervention among injured patients with alcohol dependence: who benefits from brief interventions? Drug Alcohol Depend 2010 111 13 20 10.1016/j.drugalcdep.2009.11.025 20493644 PMC2930034

[B15] McCambridge J Rollnick S Should brief interventions in primary care address alcohol problems more strongly? Addiction 2013 [Epub ahead of print] 10.1111/add.12388 PMC4153955 24433291

[B16] McCambridge J Fifty years of brief intervention effectiveness trials for heavy drinkers Drug Alcohol Rev 2011 30 567 568 10.1111/j.1465-3362.2011.00379.x 22050048

[B17] Krupski A Sears JM Joesch JM Estee S He L Dunn C Huber A Roy-Byrne P Ries R Impact of brief interventions and brief treatment on admissions to chemical dependency treatment Drug Alcohol Depend 2010 110 126 136 10.1016/j.drugalcdep.2010.02.018 20347234

[B18] Maziak W Eissenberg T Klesges RC Keil U Ward KD Adapting smoking cessation interventions for developing countries: a model for the Middle East Int J Tuberc Lung Dis 2004 8 403 413 15141730

[B19] Patel V The need for treatment evidence for common mental disorders in developing countries Psychol Med 2000 30 743 746 10.1017/S0033291799002147 11037082

[B20] van Ginneken NTP Lewin S Rao GN Meera SM Pian J Chandrashekar S Patel V Non-specialist health worker interventions for the care of mental, neurological and substance-abuse disorders in low- and middle-income countries Cochrane Database Syst Rev 2013 11 CD009149 10.1002/14651858.CD009149.pub2 24249541

[B21] Buttorff C Hock RS Weiss HA Naik S Araya R Kirkwood BR Economic evaluation of a task-shifting intervention for common mental disorders in India Bull World Health Organ 2012 90 813 821 10.2471/BLT.12.104133 23226893 PMC3506405

[B22] Padmanathan P De Silva MJ The acceptability and feasibility of task-sharing for mental healthcare in low and middle income countries: a systematic review Soc Sci Med 2013 97 82 86 24161092 10.1016/j.socscimed.2013.08.004

[B23] Tomlinson M Rudan I Saxena S Swartz L Tsai A Patel V Setting investment priorities for research in global mental health Bull World Health Organ 2009 87 438 446 10.2471/BLT.08.054353 19565122 PMC2686213

[B24] Murthy P Manjunatha N Subodh B Chand P Benegal V Substance use and addiction research in India Indian J Psychiatry 2010 52 189 199 10.4103/0019-5545.69232 PMC3146212 21836677

[B25] Patel V Araya R Chowdhary N King M Kirkwood B Nayak S Simon G Weiss HA Detecting common mental disorders in primary care in India: a comparison of five screening questionnaires Psychol Med 2008 38 221 228 18047768 10.1017/S0033291707002334 PMC4959557

[B26] Kroenke K Spitzer RL Williams JBW The PHQ-9 - Validity of a brief depression severity measure J Gen Intern Med 2001 16 606 613 10.1046/j.1525-1497.2001.016009606.x 11556941 PMC1495268

[B27] Kroenke KS Spitzer RL The PHQ-9: a new depression diagnostic and severity measure Psychiatr Ann 2002 32 91 97

[B28] Saunders JB Aasland OG Babor TF Fuente JR Grant M Development of the Alcohol Use Disorders Identification Test (AUDIT): WHO collaborative project on early detection of persons with harmful alcohol consumption-II Addiction 1993 88 791 804 10.1111/j.1360-0443.1993.tb02093.x 8329970

[B29] Babu RS Sengupta SN Sateesh Babu R A study of problem drinkers in a general hospital Indian J Psychiatry 1997 39 13 17 21584037 PMC2967075

[B30] Silva MC Gaunekar G Patel V Kukalekar DS Fernandes J The prevalence and correlates of hazardous drinking in industrial workers: a study from Goa, India Alcohol Alcohol 2003 38 79 83 10.1093/alcalc/agg016 12554613

[B31] Kunz MF French MT Bazargan-Hejazi S Cost-effectiveness analysis of a brief intervention delivered to problem drinkers presenting at an inner-city hospital emergency department J Stud Alcohol 2004 65 363 370 15222593 10.15288/jsa.2004.65.363

[B32] NICE Alcohol Dependence and Harmful Alcohol Use: Full Guideline CG115 2011 London: NICE Press Office

[B33] Fournier JC DeRubeis RJ Shelton RC Hollon SD Amsterdam JD Gallop R Prediction of response to medication and cognitive therapy in the treatment of moderate to severe depression J Consult Clin Psychol 2009 77 775 787 19634969 10.1037/a0015401 PMC2810269

[B34] Barnett NP Apodaca TR Magill M Colby SM Gwaltney C Rohsenow DJ Monti PM Moderators and mediators of two brief interventions for alcohol in the emergency department Addiction 2010 105 452 465 10.1111/j.1360-0443.2009.02814.x 20402989 PMC2858352

[B35] Fournier JC DeRubeis RJ Hollon SD Dimidjian S Amsterdam JD Shelton RC Fawcett J Antidepressant drug effects and depression severity: a patient-level meta-analysis JAMA 2010 303 47 53 10.1001/jama.2009.1943 20051569 PMC3712503

[B36] Constantino MJ Arnkoff DB Glass CR Ametrano RM Smith JZ Expectations J Clin Psychol 2011 67 184 192 10.1002/jclp.20754 21128304

[B37] Schulz KF Grimes DA Allocation concealment in randomised trials: defending against deciphering Lancet 2002 359 614 618 10.1016/S0140-6736(02)07750-4 11867132

[B38] Bower P Kontopantelis E Sutton A Kendrick T Richards DA Gilbody S Knowles S Cuijpers P Andersson G Christensen H Meyer B Huibers M Smit F van Straten A Warmerdam L Barkham M Bilich L Lovell K Liu ET Influence of initial severity of depression on effectiveness of low intensity interventions: meta-analysis of individual patient data BMJ 2013 346 f540 10.1136/bmj.f540 23444423 PMC3582703

[B39] Ekers D Richards D Gilbody S A meta-analysis of randomized trials of behavioural treatment of depression Psychol Med 2008 38 611 623 17903337 10.1017/S0033291707001614

[B40] Moradveisi L Huibers MJ Renner F Arasteh M Arntz A Behavioural activation v. antidepressant medication for treating depression in Iran: randomised trial Br J Psychiatry 2013 202 204 211 10.1192/bjp.bp.112.113696 23391727

[B41] Vasilaki EI Hosier SG Cox WM The efficacy of motivational interviewing as a brief intervention for excessive drinking: a meta-analytic review Alcohol Alcohol 2006 41 328 335 10.1093/alcalc/agl016 16547122

[B42] Bager P Vilstrup H Post-discharge brief intervention increases the frequency of alcohol abstinence—a randomized trial J Addict Nurs 2010 21 37 41 10.3109/10884601003628104

[B43] Rahman A Malik A Sikander S Roberts C Creed F Cognitive behaviour therapy-based intervention by community health workers for mothers with depression and their infants in rural Pakistan: a cluster-randomised controlled trial Lancet 2008 372 902 909 10.1016/S0140-6736(08)61400-2 18790313 PMC2603063

[B44] Hill Z Manu A Tawiah-Agyemang C Gyan T Turner K Weobong B Ten Asbroek AH Kirkwood BR How did formative research inform the development of a home-based neonatal care intervention in rural Ghana? J Perinatol 2008 28 2 S38 S45 19057567 10.1038/jp.2008.172

[B45] Rahman A Challenges and opportunities in developing a psychological intervention for perinatal depression in rural Pakistan–a multi-method study Arch Womens Ment Health 2007 10 211 219 10.1007/s00737-007-0193-9 17676431 PMC2424205

[B46] Araya R Rojas G Fritsch R Gaete J Rojas M Simon G Peters TJ Treating depression in primary care in low-income women in Santiago, Chile: a randomised controlled trial Lancet 2003 361 995 1000 10.1016/S0140-6736(03)12825-5 12660056

[B47] Dias A Dewey ME D'Souza J Dhume R Motghare DD Shaji KS Menon R Prince M Patel V The effectiveness of a home care program for supporting caregivers of persons with dementia in developing countries: a randomised controlled trial from Goa, India PLoS One 2008 3 e2333 10.1371/journal.pone.0002333 18523642 PMC2396286

[B48] Chatterjee S Chowdhary N Pednekar S Cohen A Andrew G Araya R Simon G King M Telles S Verdeli H Clougherty K Kirkwood B Patel V Integrating evidence-based treatments for common mental disorders in routine primary care: feasibility and acceptability of the MANAS intervention in Goa, India World Psychiatry 2008 7 45 53 10.1002/j.2051-5545.2008.tb00151.x PMC2359726 18458786

[B49] Verdeli H Clougherty K Bolton P Speelman L Lincoln N Bass J Neugebauer R Weissman MM Adapting group interpersonal psychotherapy for a developing country: experience in rural Uganda World Psychiatry 2003 2 114 120 16946913 PMC1525093

[B50] Bolton P Bass J Neugebauer R Verdeli H Clougherty KF Wickramaratne P Speelman L Ndogoni L Weissman M Group interpersonal psychotherapy for depression in rural Uganda: a randomized controlled trial JAMA 2003 289 3117 3124 10.1001/jama.289.23.3117 12813117

[B51] Martell CR Dimidjian S Herman-Dunn R Behavioural Activation for Depression: a Clinician’s Guide 2010 New York: The Guilford Press

[B52] Miller WR Zweben A DiClimente C Rychtarik R Motivational Enhancement Therapy Manual: A Clinical Research Guide for Therapists Treating Individuals with Alcohol Abuse and Dependence 1994 Bethesda MD: National Institute on Alcohol Abuse and Alcoholism

[B53] The PREMIUM treatment manuals http://sangath.com/manuals.php

[B54] Eriksen K McAuliffe G A measure of counselor competency Couns Educ Superv 2003 43 120 133

[B55] Allison J Bes R GR Motivational Interviewing Target Scheme (MITS 2.1) 2012 Edinburgh: Jeff Alisson Training Limited

[B56] WHO mhGA: Mental Health Gap Action Programme: Scaling up Care for Mental, Neurological and Substance Use Disorders 2008 Geneva: World Health Organization 26290926

[B57] Janakiramaiah N Gangadhar BN Naga Venkatesha Murthy PJ Harish MG Subbakrishna DK Vedamurthachar A Antidepressant efficacy of sudarshan kriya yoga (SKY) in melancholia: a randomized comparison with electroconvulsive therapy (ECT) and imipramine J Affect Disord 2000 57 255 259 10.1016/S0165-0327(99)00079-8 10708840

[B58] Kumar GS Jain A Hegde S Prevalence of depression and its associated factors using Beck Depression Inventory among students of a medical college in Karnataka Indian J Psychiatry 2012 54 223 226 10.4103/0019-5545.102412 23226844 PMC3512357

[B59] Sobell L Sobell M Timeline follow-back: a technique for assessing self-reported alcohol consumption Psychosocial and Biochemical Methods: Measuring Alcohol Consumption 1992 Totowa NJ: Humana Press 41 72

[B60] De Sousa A De Sousa A An open randomized trial comparing disulfiram and naltrexone in adolescents with alcohol dependence J Subst Use 2008 13 382 388 10.1080/14659890802305861

[B61] DeCosta G Nazareth I Naik D Vaidya R Levy G Patel V King M Patterns of harmful alcohol consumption and associated problems in general practice attendees in Goa, India Alcohol Alcohol 2007 42 131 137 17172257 10.1093/alcalc/agl103

[B62] Alterman AI Cacciola JS Ivey MA Habing B Lynch KG Reliability and validity of the alcohol SIP and a newly constructed drug short index of problems J Stud Alcohol Drugs 2009 70 304 307 19261243 10.15288/jsad.2009.70.304 PMC2653616

[B63] Bowen S Witkiewitz K Dillworth TM Chawla N Simpson TL Ostafin BD Larimer ME Blume AW Parks GA Marlatt GA Mindfulness meditation and substance use in an incarcerated population Psychol Addict Behav 2006 20 343 347 16938074 10.1037/0893-164X.20.3.343

[B64] Bedirhan Üstün T Chatterji S Kostanjsek N Rehm J Kennedy C Epping-Jordan J Saxena S von Korff M Pull C in collaboration with WHO/NIH Joint Project Developing the World Health Organization Disability Assessment Schedule 2.0 Bull World Health Organ 2010 88 815 823 10.2471/BLT.09.067231 21076562 PMC2971503

[B65] Patel V Chisholm D Kirkwood BR Mabey D Prioritizing health problems in women in developing countries: comparing the financial burden of reproductive tract infections, anaemia and depressive disorders in a community survey in India Trop Med Int Health 2007 12 130 139 17207157 10.1111/j.1365-3156.2006.01756.x

[B66] Patel V Chisholm D Rabe-Hesketh S Dias-Saxena F Andrew G Mann A Efficacy and cost-effectiveness of drug and psychological treatments for common mental disorders in general health care in Goa, India: a randomised, controlled trial Lancet 2003 361 33 39 10.1016/S0140-6736(03)12119-8 12517464

[B67] Chisholm D Sekar K Kumar KK Saeed K James S Mubbashar M Murthy RS Integration of mental health care into primary care. Demonstration cost-outcome study in India and Pakistan Br J Psychiatry 2000 176 581 588 10.1192/bjp.176.6.581 10974966

[B68] Maselko J Patel V Why women attempt suicide: the role of mental illness and social disadvantage in a community cohort study in India J Epidemiol Community Health 2008 62 817 822 10.1136/jech.2007.069351 18701733

[B69] Manos RC Kanter JW Luo W The behavioral activation for depression scale–short form: development and validation Behav Ther 2011 42 726 739 10.1016/j.beth.2011.04.004 22036000

[B70] OPSPL STAR: Sangath digital tool for advanced research 2013

[B71] Moher D Schulz FK Alman GD The CONSORT statement: revised recommendations for improving the quality of reports of parallel-group randomised trials Lancet 2001 357 1191 1194 10.1016/S0140-6736(00)04337-3 11323066

[B72] Localio AR Margolis DJ Berlin JA Relative risks and confidence intervals were easily computed indirectly from multivariable logistic regression J Clin Epidemiol 2007 60 874 882 10.1016/j.jclinepi.2006.12.001 17689803

[B73] Nur U Longford NT Cade JE Greenwood DC The impact of handling missing data on alcohol consumption estimates in the UK women cohort study Eur J Epidemiol 2009 24 589 595 10.1007/s10654-009-9384-1 19728116

[B74] Dunn G Maracy M Tomenson B Estimating treatment effects from randomized clinical trials with noncompliance and loss to follow-up: the role of instrumental variable methods Stat Methods Med Res 2005 14 369 395 10.1191/0962280205sm403oa 16178138

